# Dendritic cells in the human vaginal mucosa can direct CD4^+^ T cell responses by expressing surface OX40L

**DOI:** 10.3389/fimmu.2025.1657115

**Published:** 2025-09-02

**Authors:** HyeMee Joo, Laurie Baert, Agnes Yang, Dorothee Duluc, Johnny Yi, SangKon Oh

**Affiliations:** ^1^ Department of Immunology, Mayo Clinic, Scottsdale, AZ, United States; ^2^ Immunoconcept, CNRS UMR 5164, Bordeaux University, Bordeaux, France; ^3^ Department of Medical and Surgery Gynecology, Mayo Clinic, Phoenix, AZ, United States

**Keywords:** vaginal mucosa, female genital mucosa, Langerhans cell, dendritic cell, OX40L, Th2

## Abstract

**Introduction:**

Immunity in the vaginal mucosa (VM) is of critical importance for the protection from infections and cancers. Dendritic cells (DCs) are the major antigen-presenting cells that can induce and control T cell responses. Interestingly, VM Langerhans cells (vLCs) and VM CD1c^+^CD14^-^ DCs (vDCs) polarize CD4^+^ T cells toward Th2-type. However, the mechanisms underlying Th2 polarization by vDCs remain unknown.

**Methods:**

OX40L expression was determined in the human VM tissue sections, followed by the measurement of OX40L expression on vLCs, CD1c^+^CD14^-^ vDCs, and VM macrophages (vMØs) by flow cytometry. The roles of OX40L on vDC subsets in the induction of different types of CD4^+^ T cell responses were assessed.

**Results:**

Both vLCs and CD1c^+^CD14^-^ vDCs express surface OX40L. Neutralizing OX40L with anti-OX40L antibody significantly decreased the frequency of Th2-type CD4^+^ T cells with a reduction of CD4^+^ T cell proliferation, while increasing the frequency of IL - 10-producing CD4^+^ T cell responses. Anti-OX40L did not affect vLC- or CD1c^+^CD14^-^ vDC-induced Th1-type T cell responses. OX40L also contributed to the induction of IL - 21^+^CD4^+^ T cell responses by vLCs and CD1c^+^CD14^-^ vDCs. In contrast to vLCs and CD1c^+^CD14^-^ vDCs, vMØs expressed a minimal level of surface OX40L. Likewise, anti-OX40L did not significantly affect vMØ-induced CD4^+^ T cell responses.

**Conclusions:**

OX40L contributes to vLC- and CD1c^+^CD14^-^ vDC-induced Th2 polarization. It also significantly affects the frequency of vLC- and CD1c^+^CD14^-^ vDC-induced IL - 10^+^ and IL - 21^+^CD4^+^ T cells. This study provides new insights into the immunological landscape of the human VM tissues, with implications for the development of targeted immunomodulatory strategies at this mucosal site.

## 1 Introduction

The female genital tract, especially the vaginal mucosa (VM), has long been a site of interest for mounting mucosal immunity (1–3) for the protection of microbial infections and cancers. The human VM, a site constantly exposed to a variety of antigens and stimuli, is also a unique tissue microenvironment that can control deleterious types of immune response (1, 4–6). Despite its clinical relevance, the immunology of the human VM and female genital tract remains significantly understudied.

Dendritic cells (DCs) are the major antigen-presenting cells (APCs) that can induce and control T cell responses, directing them toward either immunity or tolerance. Understanding the biology of DCs in the human VM is therefore essential for advancing our knowledge of the immunological landscape of the human vagina and lower female genital tract.

The human VM contains four major subsets of myeloid APCs (3, 7, 8): Langerhans cells (LCs) in the epithelium, and lamina propria (LP)-resident CD1c^+^CD14^-^ DCs, CD1c^+^CD14^+^ DCs, and macrophages (MØs) that can display functional specialties at eliciting T cell responses. LCs and LP-CD1c^+^CD14^-^ DCs promote CD4^+^ T cell polarization toward Th2 phenotype, whereas MØs and CD1c^+^CD14^+^ DCs in the LP polarize CD4^+^ T cells toward Th1 phenotype. These various CD4^+^ T cell responses elicited by specific subsets of APCs are critical for mounting effective host immunity against pathogens and cancers, as well as for maintaining immune homeostasis. Both VM LCs (vLCs) and VM CD1c^+^CD14^-^ DCs (vDCs) express higher levels of co-stimulatory molecules, including CD83 and CD86, than vMØs and CD1c^+^CD14^+^ vDCs (7), supporting the potency of DCs at eliciting T cell responses. However, the mechanisms by which subsets of VM APCs (vAPCs) display functional specialties, e.g., Th2 polarization by vLCs and CD1c^+^CD14^-^ vDCs and Th1 polarization by vMØs and CD1c^+^CD14^+^ vDCs, remain to be investigated.

Human thymic stromal lymphopoietin (TSLP), an IL - 7-like cytokine, is known to be produced by epithelial cells in different organs and tissues, including lung, skin, and gut (9, 10). A growing body of evidence suggests that TSLP acts as a master switch for allergic inflammation through licensing DCs to initiate inflammatory Th2 response (11, 12). Indeed, TSLP can instruct myeloid DCs (mDCs) to prime Th2 cells by inducing surface OX40L expression by mDCs, which serves as the positive Th2-polarizing signal to directly trigger the differentiation of Th2 cells from naïve CD4^+^ T cells (11, 13, 14). OX40L expressed by TSLP-activated mDCs is also known to inhibit IL - 10 production by T cells (11, 13, 14).

Of interest, both epithelial cells and stromal cells in the female genital tract were reported to express TSLP in response to exogeneous, e.g., HIV infection (15), and endogenous stimuli, e.g., female sex hormones, estrogen (16, 17) and progesterone (18). Recently, estrogen receptor (ER) expression was noted in both epithelial and stromal cells in the human VM, whereas progesterone receptors (PRs) are mainly expressed on stromal cells in the LP of the human VM (19, 20).

In this study, we hypothesized that the magnitude and types of CD4^+^ T cell responses, especially Th2-type, elicited by vLCs and CD1c^+^CD14^-^ vDCs could be dependent on the upregulation of surface OX40L expression on their surface. This hypothesis was tested by examining the expression levels of OX40L on the vAPC subsets. We then assessed the roles of OX40L in the types and magnitude of CD4^+^ T cell responses elicited by the vAPC subsets, vLCs, CD1c^+^CD14^-^ vDCs, and vMØs, in the presence of anti-OX40L neutralizing antibody or control antibody.

## 2 Materials and methods

### 2.1 Tissue samples

Vaginal tissues were obtained from 65 patients (28 – 78 years old) who underwent pelvic or cosmetic vaginal surgeries under a protocol approved by the Institutional Review Board (IRB). Tissues were not procured from individuals who were pregnant or infected with HIV, hepatitis C virus, or tuberculosis. Tissues with severe acute inflammation (with or without microbial infections) were also excluded. Severe acute inflammation at the time of surgery was determined by surgeon’s evaluation of tissue redness, swelling, and pain. All donors were free of hormone therapy, including hormonal contraception, at the time of tissue collection. No additional diagnosis information was available. The number of tissues used in each experiment is indicated in individual figure legends. All experiments were performed in accordance with relevant guidelines and regulations.

### 2.2 Immunohistochemistry and microscopy

Cryosections were fixed in cold acetone, dried, and blocked for nonspecific fluorescence with Fc receptor blocker and background buster (Innovex Biosciences). Sections were stained with anti-OX40L (19A3, IgG2b) (14) or control antibody (IgG2b, eBioscience) and then subsequently stained with 4′,6-diamidino-2-phenylindole (DAPI) (Invitrogen). Digital images were taken using Olympus BX51 microscope utilizing the Planapo20/0.7 or Planapo40/0.95 objective, Roper Coolsnap HQ camera (Olympus) and Metamorph software (Molecular Devices). Confocal images were taken with the Leica SP1 (Leica) utilizing the Planapo63/1.32 objective. Images were acquired using the same exposures for antibody and isotype staining and identical scaling was applied.

### 2.3 vAPC isolation and staining

Tissues were cut into small pieces (approximately 1 cm^2^) and incubated in PBS containing 2 mM EDTA and antibiotic/antimycotic solution overnight at 4 °C. Epithelium and LP were then separated. LP was cut into smaller pieces (1 – 5 mm^2^). Epithelial sheets and LP pieces were incubated for 2 days at 37 °C in RPMI 1640 supplemented with HEPES buffer, L-glutamine, nonessential amino-acids, sodium pyruvate, antibiotic/antimycotic, and 10% FCS. Migratory cells from epithelium and LP were recovered, filtered consecutively on 100 μm, 70 μm and 40 μm cell strainers and washed. Cells were stained with 7-AAD, anti-HLA-DR-AF700, anti-Langerin-PE, anti-CD1c-FITC (Invitrogen) and CD14-eFluor450. HLA-DR^+^ cells were gated and then Langerin^+^ vLCs, In HLA-DR^+^Langerin^-^ cells, CD1c^+^CD14^−^ vDCs and CD1c^−^CD14^+^ vMØs (**Supplementary Figure S1**) were sorted by FACS Aria II (BD Biosciences), as previously described (7, 8). Surface OX40L and TSLP receptor (TSLPR) were stained with anti-OX40L (R&D Systems, MAB10563) and anti-TSLPR antibody (BioLegend, 1B4), respectively. In some experiments, migratory cells were cultured for 72 hours in the presence and absence of 20 ng/ml TSLP (R&D Systems, 1398-TS-010/CF).

### 2.4 T Cell responses

**Figure 1:**
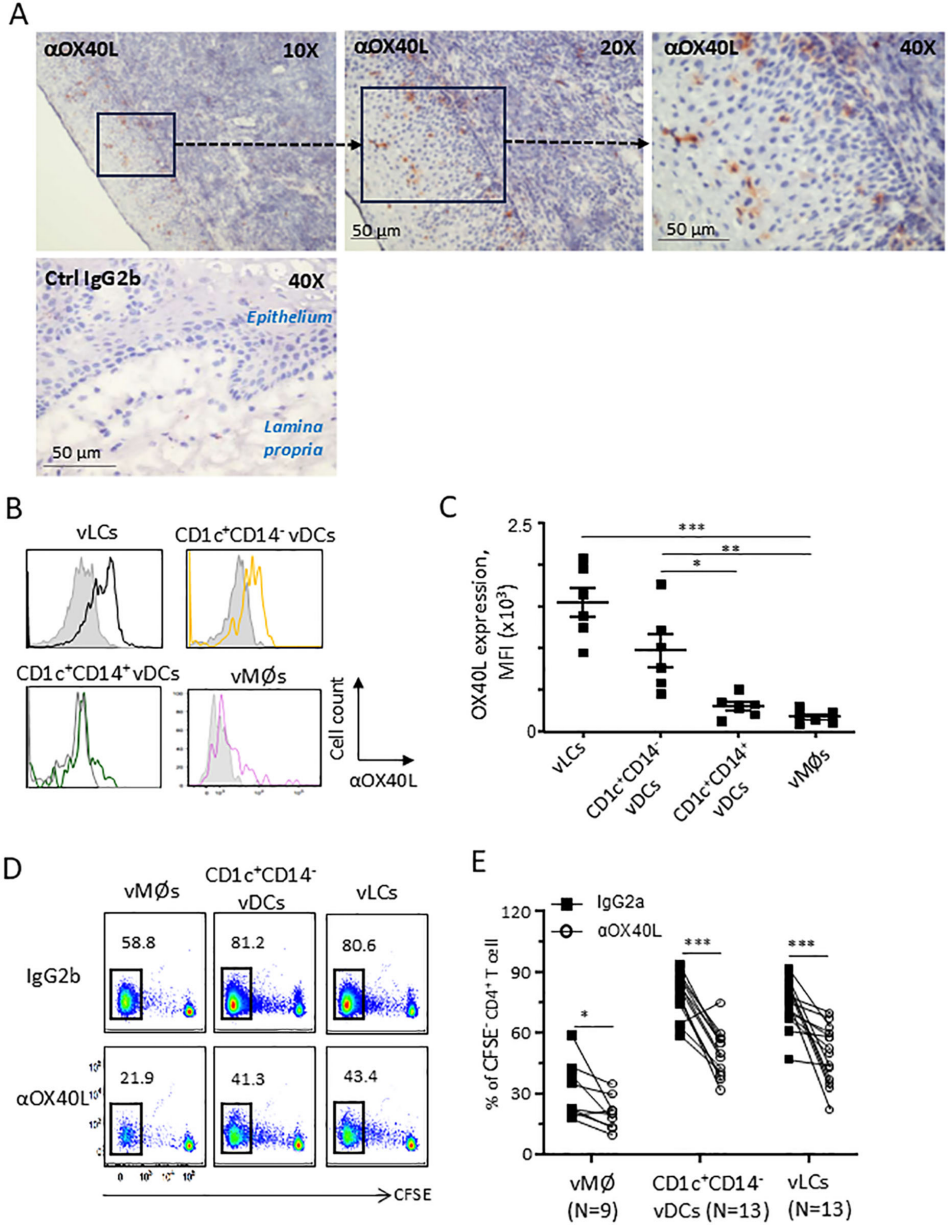
vLCs and CD1c^+^CD14^-^ vDCs express surface OX40L that contributes to CD4^+^ T cell proliferation. Frozen VM tissue sections were stained with anti-OX40L or control antibody **(A)**. Representative data generated with tissues from 4 donors are presented. Surface OX40L expression levels on vLCs, CD1c^+^CD14^-^ vDCs, CD1c^+^CD14^+^ vDCs, and vMØs were measured by flow cytometry. Representative data are presented **(B)**. Compiled data of background subtracted mean fluorescence intensity (MFI) of cells from 6 donors are presented **(C)**. FACS-sorted vLCs, CD1c^+^CD14^-^ vDCs, and vMØs were co-cultured for 6 days with purified and CFSE-labeled naïve allogeneic CD4^+^ T cells in the presence of anti-OX40L or control antibody. CD4^+^ T cell proliferation was assessed by measuring CFSE dilution with flow cytometry. Representative data **(D)** and compiled data **(E)** are presented. Statistical significance was determined using the ANOVA **(C)** and a non-parametric Wilcoxon matched-pairs signed rank test **(E)**. *P < 0.05, **P < 0.01, ***P < 0.001, ****P < 0.0001, and ns: not significant.

**Figure 2:**
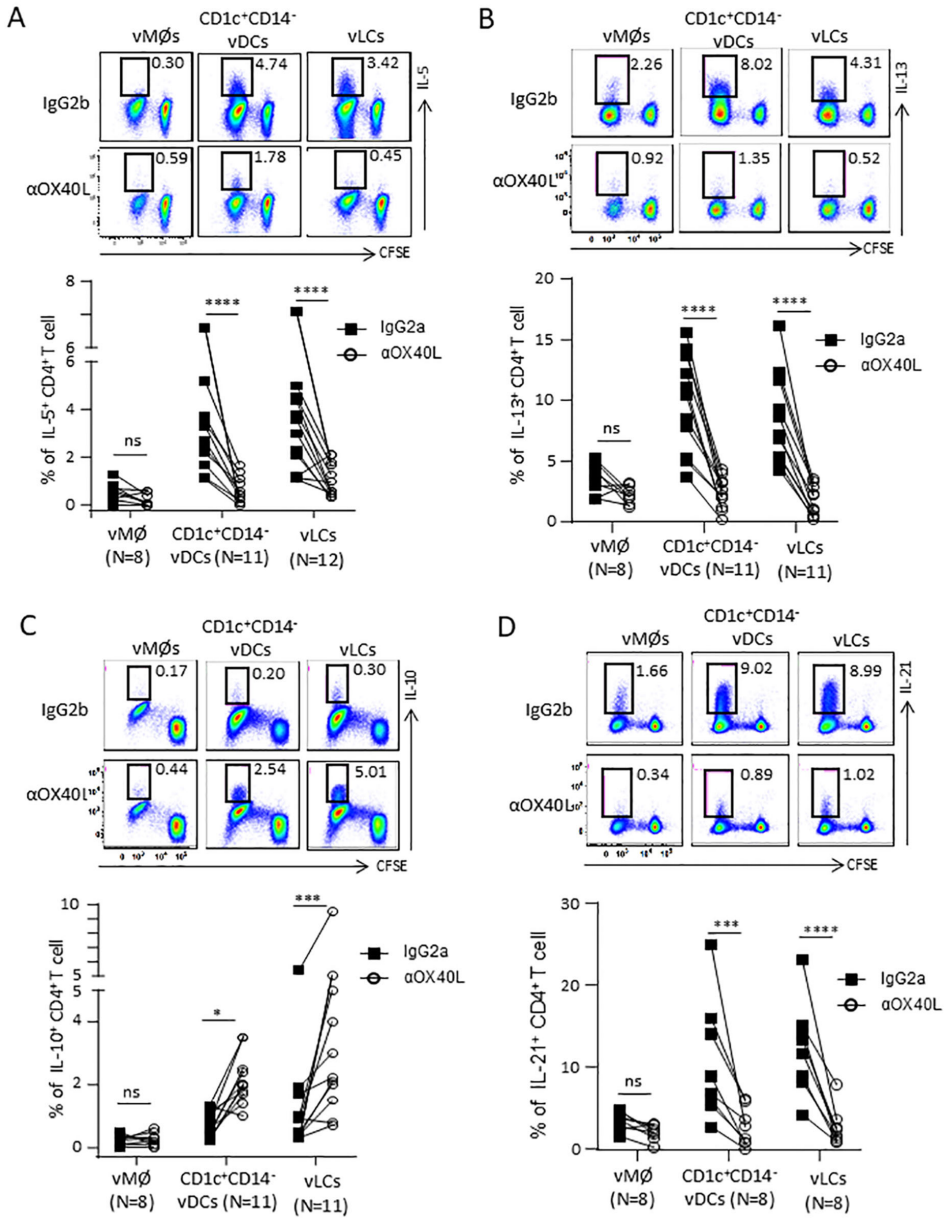
OX40L contributes to the vLC- and CD1c^+^CD14^-^ vDCs-mediated Th2 polarization. FACS-sorted vLCs, CD1c^+^CD14^-^ vDCs, and vMØs were co-cultured for 6 days with purified and CFSE-labeled naïve allogeneic CD4^+^ T cells in the presence of anti-OX40L or control antibody. T cells were restimulated with PMA/ionomycin for 5 hours in the presence of brefeldin A before staining with anti-IL-5 **(A)**, anti-IL-13 **(B)**, anti-IL-10 **(C)** and anti-IL-21 **(D)**. Representative FACS data (upper panels) and compiled data generated with cells from indicated numbers of VM tissue donors (lower panels) are presented. Statistical significance was determined using a non-parametric Wilcoxon matched-pairs signed rank test. *P < 0.05, **P < 0.01, ***P < 0.001, ****P < 0.0001, and ns: not significant.

Peripheral blood mononuclear cells (PBMCs) from 5 healthy volunteers were collected by leukapheresis under a protocol approved by the Institutional Review Board. Healthy blood donors provided informed consent in accordance with the Declaration of Helsinki. Total T cells were enriched with EasySep Human T Cell Enrichment Kit (STEMCELL, CA) according to the manufacturer’s protocol. Allogeneic naïve CD4^+^ T cells (CD45RA^+^CD45RO^–^CCR7^+^) were sorted by FACS Aria II (BD Biosciences). 1.5×10^5^ CFSE-labeled allogeneic naïve CD4^+^ T cells were co-cultured with 2×10^3^ indicated vAPC subsets (APC:T cell ratio = 1:75) in RPMI 1640 supplemented with HEPES buffer, L-glutamine, nonessential amino-acids, sodium pyruvate, penicillin/streptomycin and 10% AB serum (GemCell, CA). Experiments were performed in the presence of anti-OX40L (19A3, IgG2b) (14) or control antibody (IgG2b) (5 μg/ml). After 6 days, cells were stained with anti-CD4 APC-Cy7 (Biolegend) and LIVE/DEAD^®^ Fixable Dead Cell Stain Kit (Invitrogen), and T cell proliferation was assessed by measuring CFSE dilution. For cytokine expression analysis, T cells were restimulated with 100 ng/ml phorbol 12-myristate 13-acetate (PMA; Sigma) and 1 μg/ml ionomycin (Sigma) for 5h in the presence of GolgiPlug (BD Biosciences). They were then stained with anti-CD4 (RPA-T4, Biolegend), LIVE/DEAD^®^ Fixable Dead Cell Stain Kit (Invitrogen), anti-IFNγ (4S.B3, eBioscience), anti-TNFα (MAB210 - 100, R&D Systems), anti-IL-5 (JES1 – 39D10, Biolegend), anti-IL-13 (JES10 – 5A2, Biolegend), anti-IL-10 (JES3 – 9D7, eBioscience), or anti-IL-21 (in house (14). Intracellular staining was performed using BD Cytofix/Cytoperm™ Fixation/Permeabilization Solution Kit according to the manufacturer’s protocol. The numbers of patient donors in some experiments (i.e., **Figure 1E** and **Figure 2**) were not the same as the numbers of individual vAPC subsets that could be isolated were variable among patient donors.

### 2.5 Statistical analysis

Statistical significance was determined by the ANOVA and a non-parametric Wilcoxon matched-pairs signed rank test using the Prism 5 software (GraphPad Software Inc, CA). Significance was set at P<0.05.

## 3 Results

### 3.1 Both vLCs and CD1c^+^CD14^−^ vDCs express OX40L that can promote CD4^+^ T cell proliferation

vLCs and CD1c^+^CD14^−^ vDCs expressed higher levels of costimulatory molecules, such as CD83 and CD86, compared to vMØs (7). vLCs and CD1c^+^CD14^−^ vDCs were also more efficient than vMØs at inducing naïve CD4^+^ T cell proliferation (7). To study the mechanisms for the functional specialties of vDC subsets, we re-analyzed the transcription profiles of the human vAPC subsets (8, 19, 20). Both vLCs and CD1c^+^CD14^−^ vDCs showed a trend towards increased levels of *OX40L* mRNAs, when compared to that of vMØs, but the differences were not significant (**Supplementary Figure S2**).

As OX40L is another costimulatory molecule that can contribute to the magnitude as well as the types of CD4^+^ T cell responses (11, 13, 14), we examined protein OX40L expressions in the human VM tissues. As shown in **Figure 1A**, cells in the epithelium and LP of the frozen VM tissues expressed OX40L. We next tested whether vAPC subsets express surface OX40L by staining single cell suspensions of VM tissues with anti-OX40L antibody. After gating of vLCs, CD1c^+^CD14^−^ vDCs, CD1c^+^CD14^+^ vDCs, and vMØs, as previously reported (7, 19), we found that vLCs and CD1c^+^CD14^−^ vDCs expressed significantly higher levels of surface OX40L than vMØs and CD1c^+^CD14^+^ vDCs (**Figure 1B**). Summarized data generated with cells from 6 different tissue donors are presented in **Figure 1C**. As both vMØs and CD1c^+^CD14^+^ vDCs polarize CD4^+^ T cells toward Th1-type and they both express minimal levels of surface OX40L, we decided to use vMØs as controls for vLCs and CD1c^+^CD14^−^ vDCs in following experiments.

In support of the increased expression of OX40L on vLCs and CD1c^+^CD14^−^ vDCs (**Figures 1B, C**), they expressed higher levels of TSLP receptor (*TSLPR*) mRNA than vMØs **Supplementary Figure S3A**). However, additional TSLP did not further upregulate surface OX40L expression (**Supplementary Figure S3B**), suggesting that TSLP secreted in the VM tissues (16–18) might be sufficient to upregulate OX40L expression on both vLCs and CD1c^+^CD14^−^ vDCs.

The roles of OX40L expression on the human vAPC subsets in the induction of naïve CD4^+^ T cell proliferation was then examined. As shown in **Figure 1D** (upper panels), vLCs and CD1c^+^CD14^−^ vDCs were more efficient than vMØs at inducing allogeneic naïve CD4^+^ T cell proliferation. Blocking OX40L with anti-OX40L resulted in significantly reduced CD4^+^ T cell proliferations induced by vLCs and CD1c^+^CD14^−^ vDCs (lower panels, **Figure 1D**). Summarized data generated with cells from different tissue donors are presented in **Figure 1E**. Anti-OX40L also decreased CD4^+^ T cell proliferation induced by vMØs from some donors, but its effect was less than those observed with vLCs and CD1c^+^CD14^−^ vDCs. It was also of note that both vLCs and CD1c^+^CD14^−^ vDCs were more efficient than vMØs at inducing naïve CD4^+^ T cell proliferation even in the presence of anti-OX40L antibody, suggesting the roles of other costimulatory molecules upregulated on vLC and CD1c^+^CD14^−^ vDCs (7).

Taking these data (**Figure 1** and **Supplementary Figure S2**) together, we concluded that both vLCs and CD1c^+^CD14^−^ vDCs express increased levels of OX40L that contributes to CD4^+^ T cell proliferation.

### 3.2 OX40L directs the vLC- and CD1c^+^CD14^−^ vDC-induced Th2 polarization

OX40L expressed on TSLP-activated mDCs serves as the positive Th2-polarizing signal to directly trigger the differentiation of Th2 cells from naïve CD4^+^ T cells (11, 13, 14). Therefore, we investigated whether the increased OX40L expression on vLCs and CD1c^+^CD14^−^ vDCs similarly mediates their capacity to promote Th2-type CD4^+^ T cell responses.

As shown in **Figure 2A**, vLCs and CD1c^+^CD14^−^ vDCs induced higher frequencies of IL - 5^+^CD4^+^ T cells than vMØs. Such increases were decreased in the presence of anti-OX40L neutralizing antibody. Anti-OX40L treatment also reduced the frequency of IL - 13^+^CD4^+^ T cells (**Figure 2B**). In line with the previously published data generated with TSLP-activated blood mDCs (11, 13, 14), blocking OX40L significantly increased the frequency of IL - 10^+^CD4^+^ T cells (**Figure 2C**), while decreasing the frequency of IL - 21^+^CD4^+^ T cells (**Figure 2D**). Anti-OX40L also reduced the frequency of TNFα^+^CD4^+^ T cells (**Supplementary Figure S4A**), without altering the frequency of IFNγ^+^CD4^+^ T cells (**Supplementary Figure S4B**).

Collectively, we concluded that OX40L plays an important role in the vLC- and CD1c^+^CD14^−^ vDC-induced Th2 polarization without altering much of the frequency of IFNγ^+^CD4^+^ T cells. However, OX40L expressed on the two VM DC subsets also contributed to TNFα^+^CD4^+^ T cell responses. In addition, OX40L also promotes the vLC- and CD1c^+^CD14^−^ vDC-induced IL - 21^+^CD4^+^ T cell responses while decreasing the frequency of IL - 10-producing CD4^+^ T cells (11, 13, 14).

## 4 Discussion

This study is the first to report that the human VM tissue-resident APC subsets, especially vLCs and CD1c^+^CD14^-^ vDCs, express OX40L, which can direct their functional specialization toward Th2 polarization (7). OX40L expressed on vLCs and CD1c^+^CD14^-^ vDCs also determines the magnitude of different types of CD4^+^ T cell responses, as it promotes CD4^+^ T cell proliferation as well as the frequency of IL - 21^+^ and TNFα^+^CD4^+^ T cells, while reducing the frequency of IL - 10^+^CD4^+^ T cells.

The immunological function of OX40L expressed on the VM tissue-resident vLCs and CD1c^+^CD14^-^ vDCs turned out to be similar to that of TSLP-treated blood circulating mDCs (11, 13, 14) for the modulation of the types of CD4^+^ T cell responses. In line with the increased OX40L expression on vLCs and CD1c^+^CD14^-^ vDCs, they both expressed higher levels of *TSLPR* than vMØs. However, the addition of TSLP did not further increase OX40L expression on vLCs and CD1c^+^CD14^-^ vDCs cultured *in vitro*. This suggests that TSLP-induced OX40L expression on vLCs and CD1c^+^CD14^-^ vDCs has already been saturated in the VM tissues in which TSLP is continuously expressed by epithelial or stromal cells in response to female sex hormones (16–18). Supporting this notion, ERs are widely expressed in both the epithelial and stromal cells of the human VM, while PRs are primarily expressed in the LP of the human VM (19, 20). Given this study demonstrates the critical functions of OX40L expressed on the human VM-resident vLCs and CD1c^+^CD14^-^ vDCs, future studies should investigate whether microbial pathogens, such as HIV (15), and any other stimuli could modulate OX40L expression levels on the VM tissue-resident APC subsets. Ages and menstrual cycles could also significantly affect OX40L expression levels. Our data also suggested that TSLP secreted from cells (e.g., epithelial and stromal cells) in the female genital tracts could also play an important role in communications between vLCs/vDCs and non-immune cell types in the human VM and female genital tract, warranting further investigation.

## 5 Conclusion

This study demonstrates that OX40L expressed on vLC and CD1c^+^CD14^-^ vDCs can direct CD4^+^ T cell polarization toward Th2-type. OX40L also contributes to TNFα- and IL - 21-producing T cell responses, but it did not significantly alter IFNγ-producing T cell responses. As vMØs express low or minimal levels of OX40L, it does not significantly affect the vMØ-induced CD4^+^ T cell responses. These findings enhance our understanding of the immunology of the human VM and lower female genital tract. This study provides a foundation for the rational design of novel strategies to manipulate the types and magnitude of immune response in the female genital tract.

## Data Availability

The original contributions presented in the study are included in the article/**Supplementary Material**. Further inquiries can be directed to the corresponding author.
