# Supplementary material for: Dendritic cells in the human vaginal mucosa can direct CD4+ T cell responses by expressing surface OX40L
Source: Front Immunol. 2025 Sep 2;16:1657115. doi: 10.3389/fimmu.2025.1657115 (PMC12436386; doi:10.3389/fimmu.2025.1657115)
Supplement: Supplementary file 1 [file DataSheet1.pdf]

Supplementary Table 1. Information of study participants

Participant Demography	Age (years)[a]
Vaginal tissue donors (N=65)	64(28-78)
Hispanic (N=11)	59 (34-75)
Non-Hispanic (N=54)	67 (28-78)
White (N=47)	68 (28-78)
Black/African American (N=7)	57 (35-71)
Asian (N=3)	68 (65-71)
Others (N=8)	59 (47-74)

[a]Age is given as median (range).
