# Supplementary material for: Dendritic cells in the human vaginal mucosa can direct CD4+ T cell responses by expressing surface OX40L
Source: Front Immunol. 2025 Sep 2;16:1657115. doi: 10.3389/fimmu.2025.1657115 (PMC12436386; doi:10.3389/fimmu.2025.1657115)
Supplement: Supplementary file 2 [file DataSheet2.pdf]

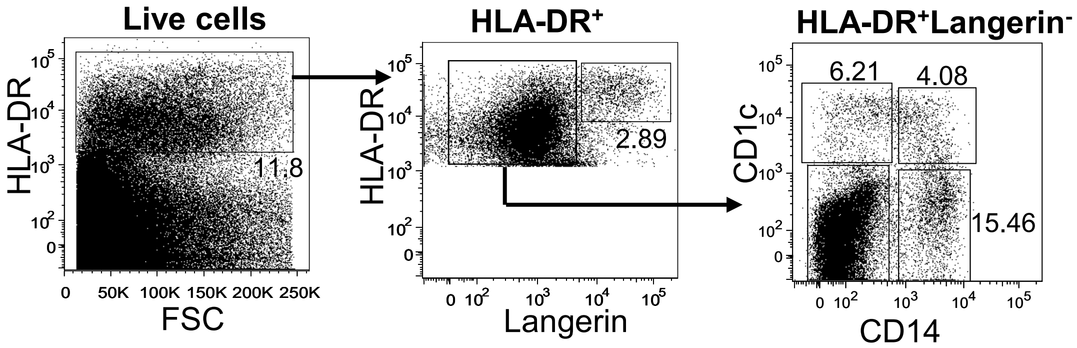

Supplementary Figure 1: Gating strategy for vLCs, CD1c⁺CD14⁻ vDCs, and CD1c⁻CD14⁺ vMØs. HLA-DR+ cells in live cells are gated. Langerin⁺ cells in HLA-DR⁺ cells are vLCs. HLA-DR⁺Langerin⁻ cells are further divided into CD1c⁺CD14⁻ vDCs, CD1c⁺ CD14⁺ vDCs, and CD1c⁻CD14⁺ vMØs. Data were generated with cells from one patient donors. The percentages of cells gated are variable among patient donors.

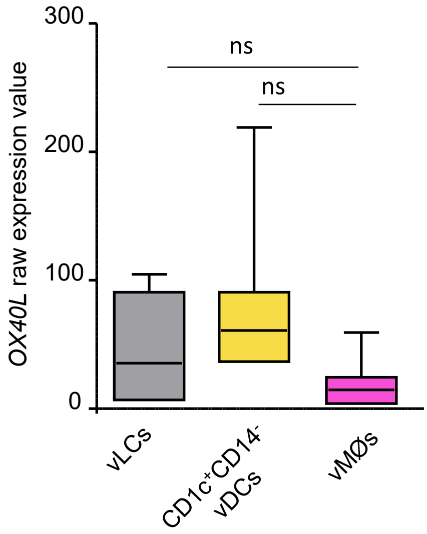

Supplementary Figure 2: vLCs and vDCs express *OX40L* mRNAs. The raw mRNA expression levels (mean ± SD) of vLCs from 9, CD1c^{+}CD14^{-} vDCs from 13, and vMØs from 14 donors, as reported in Reference 8. Statistical significance was determined using the ANOVA test. **P* < 0.05, ***P* < 0.01, ****P* < 0.001, *****P* < 0.0001, and ns: not significant.

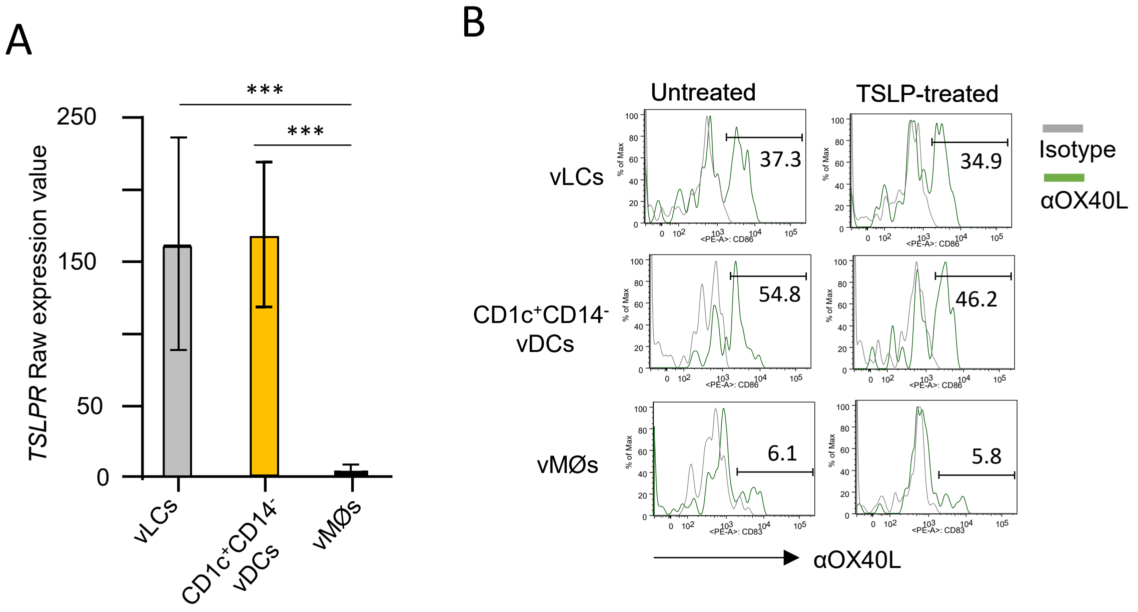

Supplementary Figure 3: vLCs and CD1c$^+$CD14$^-$ vDCs express *TSLPR,* but additional TSLP did not significantly enhance surface OX40L expression on vLCs and CD1c$^+$CD14$^-$ vDCs. (A) The raw mRNA expression levels (mean ± SD) of *TSLPR* in vLCs from 9, CD1c$^+$CD14$^-$ vDCs from 13, and vMØs from 14 donors, as reported in Reference 8. (B) Single cell suspensions of VM tissues were incubated for 72 hours in the cRPMI medium containing 20 ng/ml TSLP. Surface OX40L expression on indicated vAPC subsets were measured by flow cytometry. Data from experiments using cells from 3 donors were similar. Representative data generated with cells from one donor are presented. Statistical significance in (A) was determined using the ANOVA test. *$P < 0.05$, **$P < 0.01$, ***$P < 0.001$, ****$P < 0.0001$, and ns: not significant.

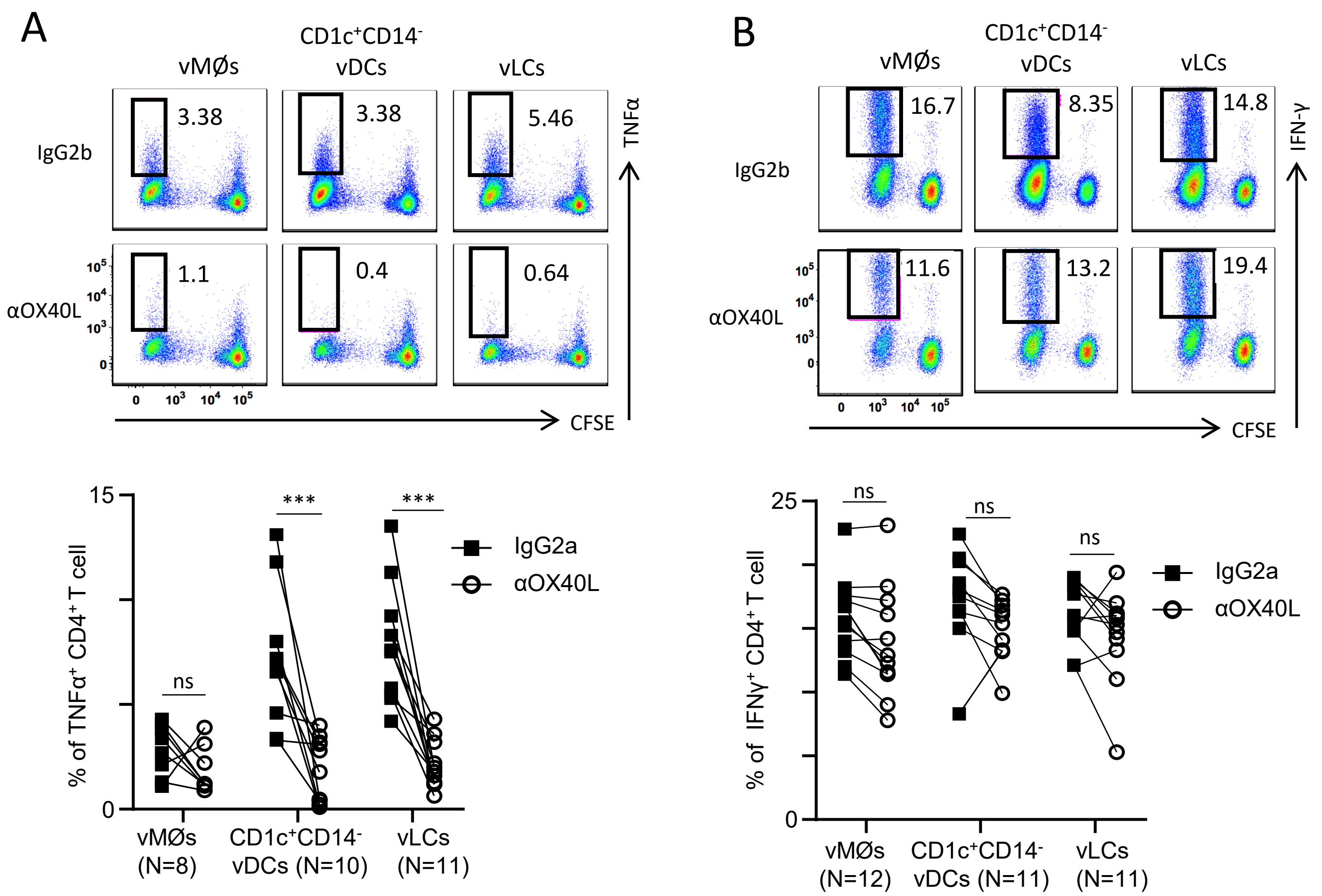

Supplementary Figure 4: OX40L expressed on vLCs and $CD1c^{+}CD14^{-}$ vDCs contributes to the induction of $TNF\alpha^{+}$ $CD4^{+}$ T cell response, but did not significantly affect $IFN\gamma^{+}$ $CD4^{+}$ T cell response. FACS-sorted vLCs, $CD1c^{+}CD14^{-}$ vDCs, and vMØs were co-cultured for 6 days with purified and CFSE-labeled naïve allogeneic $CD4^{+}$ T cells in the presence of anti-OX40L or control antibody. T cells were restimulated with PMA/ionomycin for 5 hours in the presence of brefeldin A before staining with anti-TNFα (A) and anti-IFNγ (B) antibody. Cells were gated based on isotype control antibody staining. Representative FACS data (upper panels) and compiled data generated with cells from indicated numbers of VM tissue donors (lower panels) are presented. Statistical significance was determined using a non-parametric Wilcoxon matched-pairs signed rank test. *$P < 0.05$, **$P < 0.01$, ***$P < 0.001$, ****$P < 0.0001$, and ns: not significant.
